# Mendelian Randomization Analysis Reveals Causal Effects of Circulating Metabolites on Hyperaldosteronism

**DOI:** 10.1002/fsn3.70532

**Published:** 2025-07-14

**Authors:** Chenyang Zhao, Fangjun Chen, Lixiu Peng, Qiong Li, Yajing Pang, Chaoyan Yue

**Affiliations:** ^1^ The First People's Hospital of Chenzhou Chenzhou China; ^2^ Department of Laboratory Medicine Zhongshan Hospital, Fudan University Shanghai China; ^3^ Wuxi School of Medicine Jiangnan University Wuxi Jiangsu China; ^4^ Shanghai Key Lab of Reproduction and Development, Shanghai Key Lab of Female Reproductive Endocrine Related Diseases Obstetrics and Gynecology Hospital of Fudan University Shanghai China

**Keywords:** circulating metabolites, hyperaldosteronism, mendelian randomization, metabolic analysis

## Abstract

Observational studies have demonstrated that metabolites may have effect on the development of hyperaldosteronism. However, whether these associations are causal remain uncertain. This study aimed to evaluate the causal relationships between circulating metabolites and hyperaldosteronism. We performed a two‐sample mendelian randomization (MR) analysis to investigate the causal relationships. Summary data for circulating metabolites were sourced from a large published genome‐wide association study (GWAS). Data for hyperaldosteronism were obtained from FinnGen R12, comprising 931 cases and 479,069 controls of European ancestry. The inverse‐variance weighted (IVW) method was used to estimate the causal effects. MR‐Egger regression, weighted median, and weighted mode approaches were applied as complementary analyses to assess robustness. Sensitivity analyses and reverse MR analyses were performed to evaluate the reliability of the observed associations. Additionally, pathway enrichment analysis was conducted using MetaboAnalyst 5.0. We finally identified 59 metabolites/metabolic ratios nominally associated with hyperaldosteronism (*P*_IVW_ < 0.05). Among them, 37 showed a positive and suggestive causal relationship, while 29 exhibited a negative and suggestive causal effect. Pathway analysis highlighted two significantly enriched metabolic pathways: glycerophospholipid metabolism (*p* = 4.63 × 10^−5^) and glycerolipid metabolism (*p* = 0.041). This study provides suggestive evidence for a potential influence of blood metabolites on the development of hyperaldosteronism, offering novel insights into its early detection and therapeutic strategies.

## Graphical abstract

We found 66 metabolites/metabolic ratios nominally associated with hyperaldosteronism using medenlian randomization analysis. Reverse MR analysis indicated no reverse causality between hyperaldosteronism and the 1400 metabolites/metabolite ratios. Metabolomic pathway analysis revealed that two main pathways are involved in the impact of metabolites on hyperaldosteronism.

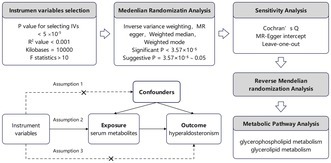

## 1 Introduction

Hyperaldosteronism is a frequent cause of secondary hypertension, affecting approximately 5% ~ 10% of hypertensive patients, and is associated with target organ involvement and damage (Funder et al. 2016). Hyperaldosteronism is characterized by the overproduction of aldosterone, primarily due to aldosterone‐producing adenomas or bilateral adrenal hyperplasia, and is termed endocrine hypertension. Previous studies have shown that hyperaldosteronism not only causes elevated arterial blood pressure, but also increases the risk of insulin resistance, type 2 diabetes, osteoporosis, cardiovascular, and cerebrovascular complications in patients with primary aldosteronism, compared to the general population and individuals with essential hypertension (Fallo et al. 2006, 2012; Hanslik et al. 2015). This indicates that hyperaldosteronism has a significant impact on human health and has increasingly become a focus of global public health efforts. Early diagnosis and timely treatment can help reduce the adverse effects of the disease on patients. However, current diagnostic procedures for hyperaldosteronism are not only costly but also complex, often requiring multiple follow‐up tests to confirm the diagnosis. This places a considerable financial and psychological burden on patients. Moreover, the underlying pathophysiological mechanisms of hyperaldosteronism remain incompletely understood, which hinders the development of effective therapeutic strategies.

With advancements in metabolomics, there is growing evidence suggesting a potential connection between metabolic factors and the development of hyperaldosteronism. For instance, targeted metabolomics studies have indicated that various metabolites are linked with secondary hypertension, including hyperaldosteronism. Metabolites derived from amino acid metabolism, such as amino acids and biogenic amines, not only hold diagnostic predictive value for hyperaldosteronism but may also be implicated in its pathogenesis (Erlic et al. 2021). A cross‐sectional study demonstrated elevated levels of branched‐chain amino acids, trimethylamine N‐oxide, and betaine in the blood of hyperaldosteronism patients (Kittithaworn et al. 2024). Further metabolomics analysis revealed that, alongside amino acids, levels of C18:2, spermidine, and phosphatidylcholine diacyl C42:4, as well as the ratios of CPT‐I, spermidine/putrescine, and total DMA/arginine, were increased, indicating an association with aldosterone secretion in hyperaldosteronism (Knuchel et al. 2024). However, these findings stem from observational studies, which are subject to limitations such as confounding variables like gender, age, BMI, or reverse causality (Sekula et al. 2016). More studies are required to establish a causal relationship between these metabolites and hyperaldosteronism, providing new insights that could aid in mechanistic understanding, early diagnosis, and therapeutic approaches for hyperaldosteronism.

Mendelian randomization (MR) is a robust epidemiological method that utilizes genetic variation as an instrumental variable (IV) to evaluate the causal relationship between an exposure and an outcome (Lawlor et al. 2008). This technique offers several advantages over traditional observational studies. Firstly, since the allocation of DNA during meiosis follows a random distribution, MR studies are less prone to confounding biases. Secondly, because the genome remains stable throughout an individual's life, MR results are less influenced by reverse causality (Burgess et al. 2015). Therefore, we conducted a bidirectional MR analysis using two samples to explore the causal relationships between serum metabolites and hyperaldosteronism, aiming to identify specific metabolites that may contribute to the development of hyperaldosteronism.

## 2 Methods

### 2.1 Study of Design

**FIGURE 1:**
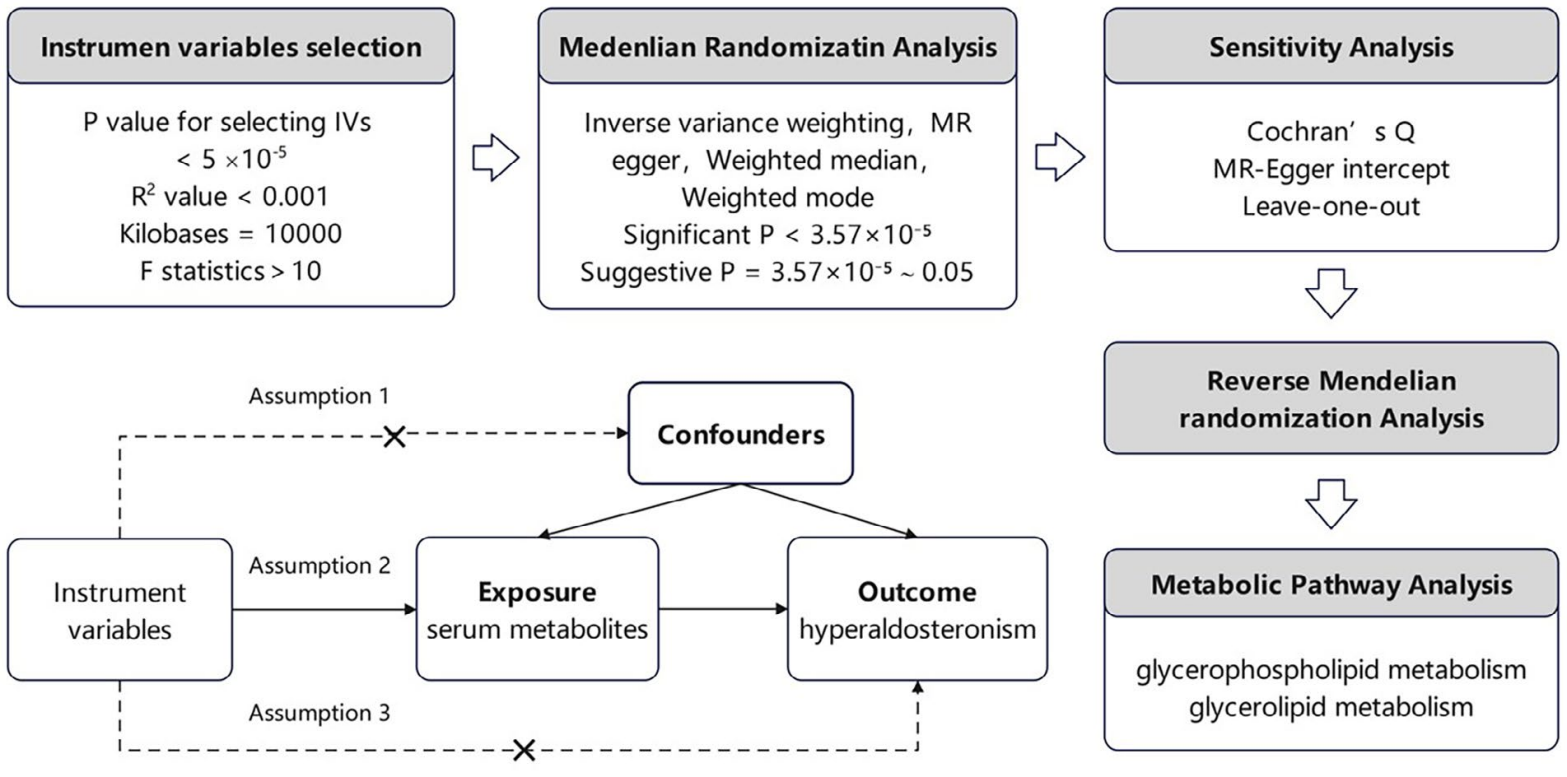
The flowchart of the study design.

We conducted a two‐sample bidirectional MR analysis to evaluate the causal relationships between 1400 metabolites/metabolite ratios and hyperaldosteronism. Additionally, we performed metabolomics analysis based on the MR results to investigate the metabolic pathways by which metabolites influence hyperaldosteronism. The MR analysis adhered to three fundamental assumptions: (1) a strong correlation exists between the IV and the exposure (relevance assumption), (2) the IV is independent of any confounders that might affect the exposure‐outcome relationship (independence assumption), and (3) the IV influences the outcome solely through its effect on the exposure (exclusion restriction assumption). This study was conducted and reported in strict adherence to the STROBE‐MR guidelines (Skrivankova et al. 2021). The flow chart of this study is illustrated in Figure 1.

### 2.2 Data Source

Summary statistics on the association between single nucleotide polymorphisms (SNPs) and serum metabolites were sourced from a large published genome‐wide association study (GWAS), encompassing 1400 serum metabolites/metabolite ratios divided into eight primary metabolic categories: amino acids, carbohydrates, cofactors and vitamins, energy metabolism, lipids, nucleotides, peptides, and xenobiotics (Chen et al. 2023). Pooled data on SNPs associated with hyperaldosteronism were obtained from FinnGen R12, comprising 931 cases (identified using ICD‐8, 9, and 10 code E26 from registry data) and 479,069 controls. All hyperaldosteronism participants in the study were of European ancestry. Since the data from this study are publicly available, no specific ethical consent or review was required from the participants of the aforementioned GWAS.

### 2.3 IV Selection

To ensure the validity of the IV, the selection of the IV must meet the three key assumptions of MR. Therefore, we established rigorous screening criteria. Initially, considering the limited number of SNPs reaching genome‐wide significance for metabolites, we adjusted the threshold by setting the *p* value to less than 1 × 10^−5^ for selecting IVs and excluding those associated with the outcome. For each serum metabolite, we set the linkage disequilibrium (LD) threshold at *r* ^2^ < 0.001, with a genetic window of 10,000 kb to exclude highly correlated SNPs and retain only independent SNPs. Additionally, to reduce the bias introduced by weak IVs, we calculated the *F*‐value for each SNP and retained only those with *F*‐values greater than 10 as strong IVs. The *F*‐value was calculated using the formula: *F* = *R* ^2^ × (*N* − 2)/(1 − *R* ^2^), where *R* ^2^ represents the proportion of variance explained by the selected SNP, and N denotes the sample size of the GWAS (Burgess and Thompson 2011).

### 2.4 MR Analysis

In our MR analyses, we employed traditional fixed‐effects inverse variance weighting (IVW) methods to estimate the causal effect of exposure on the outcome (Burgess et al. 2013). For MR analyses exhibiting high variance heterogeneity as measured by Cochran's *Q* statistic, we applied random‐effects IVW methods to adjust for heterogeneity (Greco et al. 2015). The IVW method yields the most reliable estimates when the genetic variants adhere to the three basic assumptions and there is no pleiotropy. To strengthen the robustness of our findings, we utilized three additional MR methods: MR‐Egger, weighted median, and weighted mode. The MR‐Egger regression is capable of detecting and adjusting for horizontal pleiotropy, thereby offering more reliable causal effect estimates under such conditions. The weighted median method can exclude up to 50% of IVs, thus providing more robust results in the presence of some heterogeneity. We also applied Cochran's *Q* test for heterogeneity to assess the consistency of the SNPs, with a *p* value greater than 0.05 indicating the absence of heterogeneity. The MR‐Egger intercept was used to detect horizontal pleiotropy, with a *p* value greater than 0.05 indicating its absence.

To further validate our results, we conducted leave‐one‐out analyses to check if individual SNPs influenced the overall causal relationship. We used odds ratio (OR) and 95% confidence interval (CI) to quantify the association between exposures and outcomes. A metabolite was considered to have suggestive evidence of causality with hyperaldosteronism if the IVW *p* value was less than 0.05, all four MR methods showed a consistent direction of causal effect, the correct causal direction was confirmed by the Steiger test (Table S1), and heterogeneity and horizontal pleiotropy were ruled out. All analyses were conducted using the TwoSampleMR R package in R software version 4.3.0.

### 2.5 Metabolic Pathway Analysis

Metabolites screened as positive through MR methods were included in the metabolic pathway analysis based on the KEGG and HMDB databases. The corresponding metabolite IDs were initially searched for in the Human Metabolome Database. To explore potential metabolite clusters or pathways that may be associated with the biological processes of hyperaldosteronism, metabolic pathway analysis was conducted using the MetaboAnalyst 5.0 (https://www.metaboanalyst.ca/) enrichment analysis module.

## 3 Results

### 3.1 Overall MR Results

The analysis involved 480,000 individuals of European ancestry, including 931 hyperaldosteronism cases and 479,069 healthy controls. In this study, we exclusively reported results for positive metabolites. The number of SNPs for all positive metabolites ranged from 11 to 85, and the *F*‐statistics for the validity tests of genetic predictors for all positive metabolites exceeded 10. Results from the four MR methods identified 49 serum metabolites and 17 metabolic ratios associated with hyperaldosteronism (*P*_IVW_ < 0.05, with consistent direction across all four methods). The structures and functions of the seven metabolites have not yet been defined, including X‐11852, X‐11849, X‐12410, X‐12816, X‐13431, X‐12117, and X‐17438. 59 well‐defined metabolites/metabolic ratios derived from various metabolic pathways, including amino acid, lipid, carbohydrate, and vitamin metabolism. Among these, 37 metabolites/metabolic ratios showed a positive and suggestive causal relationship with hyperaldosteronism (OR > 1), while 29 exhibited a negative and suggestive causal effect (OR < 1).

### 3.2 Association of Serum Metabolites With Hyperaldosteronism

**FIGURE 2:**
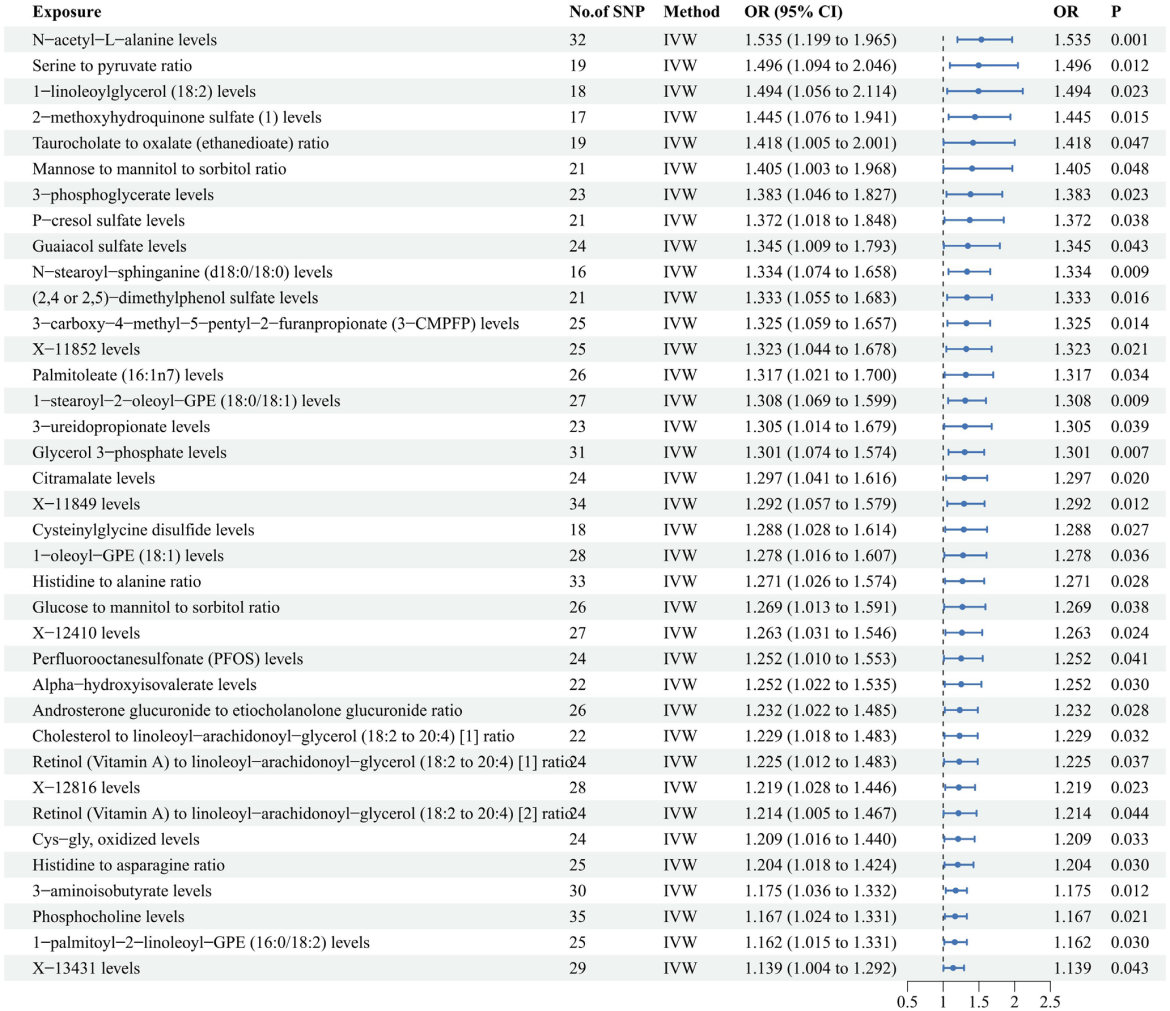
Forest plot to visualize the positive and suggestive causal associations between 37 circulating metabolites/metabolic ratios and hyperaldosteronism. CI, confidence interval; OR, odds ratio; SNP, single nucleotide polymorphism.

Figure 2 presents the 32 well‐defined metabolites/metabolic ratios and five undefined metabolites that showed a positive and suggestive causal relationship with hyperaldosteronism using the IVW method. The top 10 well‐defined metabolite/metabolite ratios with the highest ORs from the IVW analysis include: N‐acetyl‐L‐alanine levels (OR = 1.535, 95% CI: 1.119–1.965, *p* = 0.001), Serine to pyruvate ratio (OR = 1.496, 95% CI: 1.094–2.046, *p* = 0.012), 1‐linoleoylglycerol (18:2) levels (OR = 1.494, 95% CI: 1.056–2.114, *p* = 0.023), 2‐methoxyhydroquinone sulfate levels (OR = 1.445, 95% CI: 1.076–1.941, *p* = 0.015), Taurocholate to oxalate (ethanedioate) ratio (OR = 1.418, 95% CI: 1.005–2.001, *p* = 0.047), Mannose to mannitol to sorbitol ratio (OR = 1.405, 95% CI: 1.003–1.968, *p* = 0.048), 3‐phosphoglycerate levels (OR = 1.383, 95% CI: 1.046–1.827, *p* = 0.023), P‐cresol sulfate levels (OR = 1.372, 95% CI: 1.018–1.848, *p* = 0.038), Guaiacol sulfate levels (OR = 1.345, 95% CI: 1.009–1.793, *p* = 0.043), and N‐stearoyl‐sphinganine (d18:0/18:0) levels (OR = 1.334, 95% CI: 1.074–1.658, *p* = 0.009).

**FIGURE 3:**
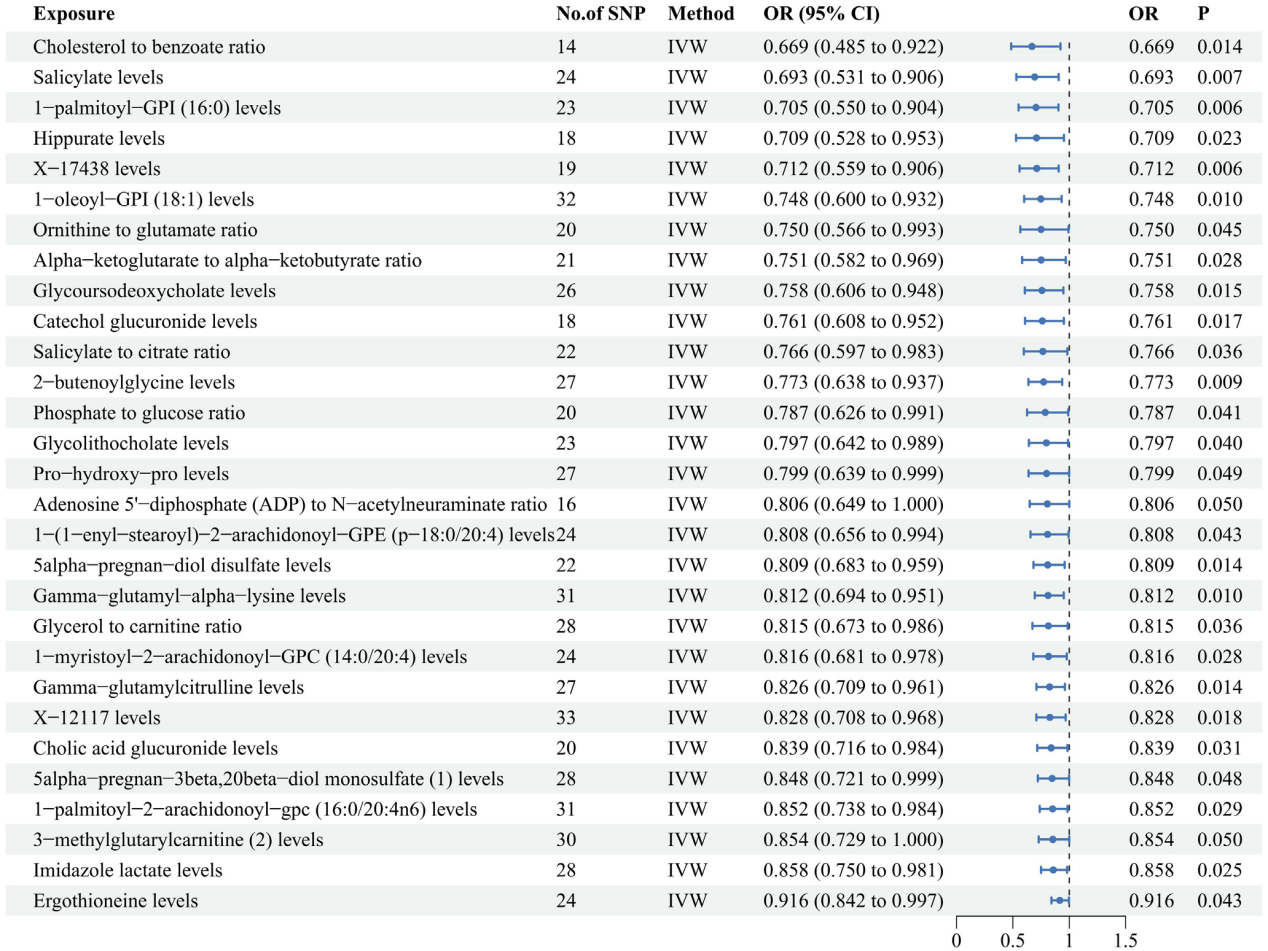
Forest plot to visualize the negative and suggestive associations between 29 circulating metabolites/metabolic ratios and hyperaldosteronism. CI, confidence interval; OR, odds ratio; SNP, single nucleotide polymorphism.

Figure 3 shows the 27 well‐defined metabolites/metabolic ratios and two undefined metabolites that exhibited a negative and suggestive causal relationship with hyperaldosteronism using the IVW method. The top 10 well‐defined metabolite/metabolite ratios with the lowest ORs from the IVW analysis include: Cholesterol to benzoate ratio (OR = 0.669, 95% CI: 0.485–0.922, *p* = 0.014), Salicylate levels (OR = 0.693, 95% CI: 0.531–0.906, *p* = 0.007), 1‐palmitoyl‐GPI (16:0) levels (OR = 0.705, 95% CI: 0.550–0.904, *p* = 0.006), Hippurate levels (OR = 0.709, 95% CI: 0.528–0.953, *p* = 0.023), X‐17438 levels (OR = 0.712, 95% CI: 0.559–0.906, *p* = 0.006), 1‐oleoyl‐GPI (18:1) levels (OR = 0.748, 95% CI: 0.600–0.932, *p* = 0.010), Ornithine to glutamate ratio (OR = 0.750, 95% CI: 0.566–0.993, *p* = 0.045), Alpha‐ketoglutarate to alpha‐ketobutyrate ratio (OR = 0.751, 95% CI: 0.582–0.969, *p* = 0.028), Glycoursodeoxycholate levels (OR = 0.758, 95% CI: 0.606–0.948, *p* = 0.015), and Catechol glucuronide levels (OR = 0.761, 95% CI: 0.608–0.952, *p* = 0.017).

The results from the three additional MR methods (MR‐Egger, Weighted mode, and Weighted median) were consistent with the IVW findings in terms of both direction and magnitude of the causal effect (Table S2). However, when applying Bonferroni correction (*p* < 0.05/1400 ≈ 3.57 × 10^−5^) as the significance threshold for causality, no metabolites met this criterion. All metabolites showing suggestive causal relationships only reached nominal significance.

Sensitivity analyses revealed that none of the 66 metabolites/metabolic ratios demonstrated heterogeneity or horizontal pleiotropy (*p* > 0.05) (Table 1). Leave‐one‐out analysis indicated that no single independent SNP significantly influenced the MR results.

**TABLE 1:** Sensitivity analysis for the associations between circulating metabolites/metabolites ratios and hyperaldosteronism.

Metabolites	Cochran's *Q* test				MR‐Egger intercept		
	*Q* of MR egger	*p* of MR egger	*Q* of IVW	*p* of IVW	Intercept	SE	*p*
N‐acetyl‐L‐alanine levels	34.434	0.264	34.518	0.303	0.009	0.035	0.789
Serine to pyruvate ratio	26.174	0.071	26.176	0.096	0.002	0.051	0.971
1‐linoleoylglycerol (18:2) levels	17.973	0.325	18.872	0.336	−0.048	0.054	0.384
2‐methoxyhydroquinone sulfate (1) levels	8.422	0.906	8.601	0.929	−0.019	0.044	0.678
Taurocholate to oxalate (ethanedioate) ratio	22.980	0.150	23.202	0.183	0.025	0.063	0.690
Mannose to mannitol to sorbitol ratio	28.050	0.082	28.295	0.103	0.024	0.058	0.688
3‐phosphoglycerate levels	13.584	0.887	14.381	0.887	−0.035	0.04	0.382
P‐cresol sulfate levels	15.205	0.709	16.743	0.670	−0.050	0.04	0.230
Guaiacol sulfate levels	21.437	0.494	25.91	0.305	−0.068	0.032	0.046
N‐stearoyl‐sphinganine (d18:0/18:0) levels	14.343	0.425	15.663	0.405	−0.031	0.027	0.275
(2,4 or 2,5)‐dimethylphenol sulfate levels	20.739	0.351	21.096	0.392	0.026	0.046	0.575
3‐carboxy‐4‐methyl‐5‐pentyl‐2‐furanpropionate (3‐CMPFP) levels	13.371	0.943	13.506	0.957	−0.011	0.03	0.716
Palmitoleate (16:1n7) levels	23.824	0.472	23.952	0.522	−0.013	0.037	0.724
1‐stearoyl‐2‐oleoyl‐GPE (18:0/18:1) levels	29.848	0.230	30.573	0.245	−0.024	0.030	0.443
3‐ureidopropionate levels	15.253	0.810	16.321	0.800	0.031	0.03	0.313
Glycerol 3‐phosphate levels	27.585	0.54	28.752	0.531	−0.03	0.027	0.289
Citramalate levels	21.390	0.497	23.995	0.404	−0.049	0.031	0.121
Cysteinylglycine disulfide levels	18.587	0.291	20.398	0.254	−0.045	0.036	0.230
1‐oleoyl‐GPE (18:1) levels	20.037	0.790	20.322	0.817	−0.019	0.035	0.598
Histidine to alanine ratio	35.037	0.282	35.088	0.324	−0.005	0.024	0.833
Glucose to mannitol to sorbitol ratio	23.258	0.505	24.116	0.513	0.029	0.031	0.364
Perfluorooctanesulfonate (PFOS) levels	18.870	0.653	20.123	0.634	0.037	0.033	0.275
Alpha‐hydroxyisovalerate levels	18.925	0.527	18.962	0.588	0.006	0.03	0.849
Androsterone glucuronide to etiocholanolone glucuronide ratio	17.696	0.817	17.913	0.846	−0.011	0.023	0.645
Cholesterol to linoleoyl‐arachidonoyl‐glycerol (18:2 to 20:4) [1] ratio	28.785	0.092	28.967	0.115	0.010	0.029	0.726
Retinol (vitamin A) to linoleoyl‐arachidonoyl‐glycerol (18:2 to 20:4) [1] ratio	16.907	0.768	20.108	0.635	−0.052	0.029	0.087
Retinol (vitamin A) to linoleoyl‐arachidonoyl‐glycerol (18:2 to 20:4) [2] ratio	22.521	0.429	22.53	0.489	−0.003	0.033	0.929
Cys‐gly, oxidized levels	22.463	0.433	24.711	0.365	−0.034	0.023	0.152
Histidine to asparagine ratio	16.520	0.832	16.534	0.868	−0.002	0.02	0.907
3‐aminoisobutyrate levels	31.073	0.314	31.155	0.358	−0.005	0.017	0.787
Phosphocholine levels	38.581	0.232	38.626	0.268	−0.004	0.019	0.846
1‐palmitoyl‐2‐linoleoyl‐GPE (16:0/18:2) levels	24.153	0.395	25.401	0.384	−0.025	0.023	0.287
Cholesterol to benzoate ratio	9.947	0.621	10.420	0.659	0.037	0.054	0.505
Hippurate levels	9.333	0.899	9.416	0.926	−0.010	0.036	0.777
Ornithine to glutamate ratio	18.146	0.446	18.277	0.504	−0.017	0.047	0.722
Alpha‐ketoglutarate to alpha‐ketobutyrate ratio	8.855	0.976	12.152	0.911	0.053	0.029	0.085
Glycoursodeoxycholate levels	21.649	0.600	21.976	0.637	−0.016	0.028	0.572
Catechol glucuronide levels	12.377	0.718	12.687	0.757	0.026	0.046	0.586
Salicylate to citrate ratio	12.328	0.904	12.380	0.929	0.008	0.037	0.821
Phosphate to glucose ratio	13.161	0.782	13.257	0.825	−0.008	0.025	0.761
Glycolithocholate levels	20.006	0.521	20.006	0.583	−0.001	0.029	0.981
Pro‐hydroxy‐pro levels	14.853	0.945	14.979	0.958	−0.009	0.026	0.725
Adenosine 5′‐diphosphate (ADP) to N‐acetylneuraminate ratio	10.18	0.749	10.57	0.782	−0.023	0.037	0.543
1‐(1‐enyl‐stearoyl)‐2‐arachidonoyl‐GPE (p‐18:0/20:4) levels	20.305	0.564	21.099	0.575	0.029	0.032	0.382
5alpha‐pregnan‐diol disulfate levels	15.401	0.753	15.412	0.802	−0.004	0.040	0.918
Glycerol to carnitine ratio	17.622	0.889	18.116	0.900	−0.015	0.021	0.488
1‐myristoyl‐2‐arachidonoyl‐GPC (14:0/20:4) levels	15.439	0.843	16.133	0.850	−0.020	0.024	0.414
Gamma‐glutamylcitrulline levels	31.091	0.186	31.652	0.205	0.014	0.021	0.508
Cholic acid glucuronide levels	15.100	0.655	15.391	0.697	0.017	0.031	0.596
5alpha‐pregnan‐3beta,20beta‐diol monosulfate (1) levels	17.982	0.876	18.45	0.889	0.016	0.024	0.500
1‐palmitoyl‐2‐arachidonoyl‐gpc (16:0/20:4n6) levels	39.971	0.084	42.822	0.061	−0.031	0.022	0.161
3‐methylglutarylcarnitine (2) levels	19.505	0.882	19.770	0.900	−0.012	0.024	0.611
Imidazole lactate levels	22.259	0.674	22.264	0.724	0.002	0.022	0.943
Ergothioneine levels	20.067	0.579	20.441	0.615	0.011	0.018	0.547
Gamma‐glutamyl‐alpha‐lysine levels	27.071	0.568	29.377	0.498	0.029	0.019	0.140
2‐butenoylglycine levels	21.979	0.637	22.386	0.667	−0.025	0.04	0.529
1‐oleoyl‐GPI (18:1) levels	22.746	0.826	23.711	0.822	−0.031	0.032	0.334
1‐palmitoyl‐GPI (16:0) levels	16.693	0.730	18.521	0.675	−0.041	0.030	0.191
Salicylate levels	13.010	0.933	13.924	0.929	0.032	0.034	0.349
X‐11852 levels	28.488	0.198	28.654	0.233	−0.012	0.032	0.717
X‐11849 levels	28.397	0.650	28.568	0.688	0.013	0.032	0.682
X‐12410 levels	15.126	0.938	15.142	0.955	0.004	0.029	0.899
X‐12816 levels	21.543	0.713	21.621	0.756	0.007	0.026	0.783
X‐13431 levels	24.727	0.590	25.443	0.604	−0.020	0.024	0.405
X‐12117 levels	26.340	0.705	26.380	0.747	0.004	0.020	0.843
X‐17438 levels	21.803	0.192	21.857	0.238	0.008	0.040	0.840

### 3.3 Association of Hyperaldosteronism With Serum Metabolites

We conducted a bidirectional MR analysis to evaluate whether there was reverse causality between hyperaldosteronism and 1400 metabolites/metabolite ratios. The IVW method served as the primary analytical approach, while the other three methods were used as complementary methods. The results from all four methods indicated no evidence of reverse causality between genetically predicted hyperaldosteronism and 1400 metabolites/metabolite ratios (*p* > 0.05). These findings are detailed in Table S3.

### 3.4 Metabolomics Analysis

**FIGURE 4:**
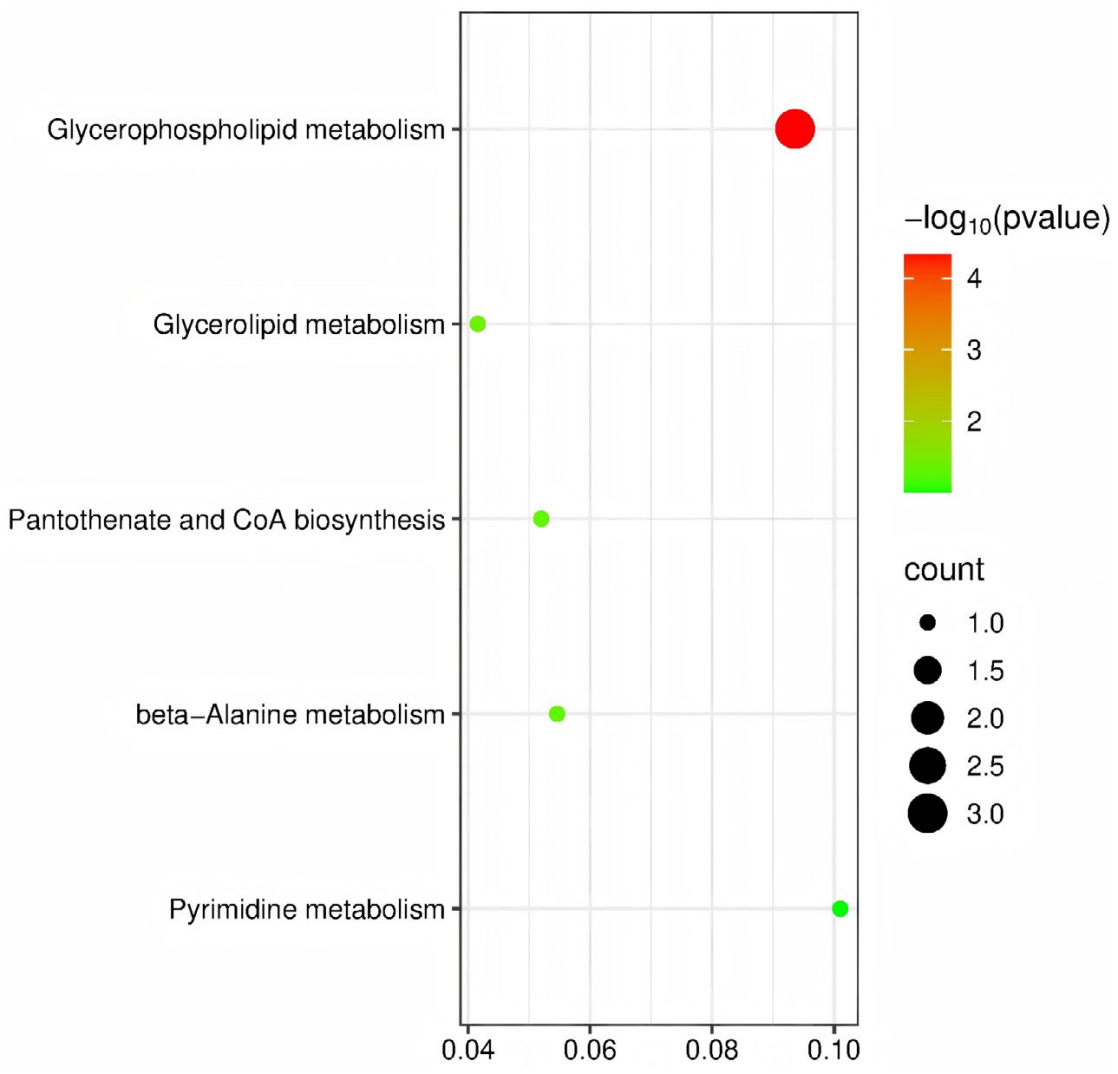
Enriched metabolic pathways of the 36 candidate metabolites.

We performed metabolic pathway analysis on the 42 metabolites with known structures and suggestive causal relationships. Using the KEGG and HMDB databases, we successfully mapped 36 of these metabolites. As shown in Figure 4, two metabolic pathways were identified as potentially associated with hyperaldosteronism: glycerophospholipid metabolism (*p* = 4.63 × 10^−5^) and glycerolipid metabolism (*p* = 0.041). Therefore, we hypothesize that the metabolic mechanisms involving these metabolites may play a role in the onset and progression of hyperaldosteronism.

## 4 Discussion

We conducted a two‐sample bidirectional MR study and metabolomics analysis to explore the causal relationships between 1400 human serum metabolites/metabolite ratios and hyperaldosteronism, as well as the biological processes by which these metabolites might influence hyperaldosteronism. Among the 59 metabolites nominally associated with hyperaldosteronism using the IVW method, 32 showed a positive association, with the most significant being N‐acetyl‐L‐alanine (OR = 1.535, 95% CI: 1.119–1.965). The remaining 27 showed a negative association, with the most significant being the cholesterol to benzoate ratio (OR = 0.669, 95% CI: 0.485–0.922). Additionally, reverse MR analysis indicated no reverse causality between hyperaldosteronism and the 1400 metabolites/metabolite ratios. Metabolomic pathway analysis revealed that two main pathways are involved in the impact of metabolites on hyperaldosteronism. Specifically, glycerophospholipid metabolism was significantly associated with the pathogenesis of hyperaldosteronism (*p* = 4.63 × 10^−5^), while glycerolipid metabolism also showed some relevance (*p* = 0.041). Therefore, we suggest that metabolic mechanisms involving these pathways may play potential roles in the development and progression of hyperaldosteronism.

Previous observational studies on metabolites and hyperaldosteronism were limited. However, with the advancement of metabolomics, researchers have identified abnormalities in metabolite levels in the blood or body fluids of hyperaldosteronism. One cross‐sectional study noted elevated levels of branched‐chain amino acids (BCAAs), such as leucine, isoleucine, and valine, in hyperaldosteronism (Kittithaworn et al. 2024). Indeed, several studies have demonstrated a positive association between BCAA concentrations in the blood and the risk of developing hypertension and cardiovascular and metabolic diseases (Würtz et al. 2013; Ruiz‐Canela et al. 2016; Flores‐Guerrero et al. 2019). Although our study did not find a causal relationship between branched‐chain amino acids and hyperaldosteronism, we observed that several amino acid‐related metabolites were associated with altered risk of hyperaldosteronism. Specifically, N‐acetyl‐L‐alanine, 3‐aminoisobutyrate, Cys‐gly, oxidized, and phosphocholine (PC) were linked to an increased risk of hyperaldosteronism. In contrast, gamma‐glutamylcitrulline and gamma‐glutamyl‐alpha‐lysine were associated with a decreased risk. To further explore the potential biological relevance of these findings, we conducted pathway enrichment analysis. The results suggested that amino acid metabolism pathways may be involved in the pathogenesis of hyperaldosteronism, although the associations did not reach statistical significance after correction (*p* = 0.053). Nonetheless, these findings may provide some suggestive insights into the metabolic mechanisms underlying hyperaldosteronism.

There is a diverse range of amino acids present in living organisms, and though direct studies linking amino acids to hyperaldosteronism are relatively scarce, amino acids are known to be closely associated with the development of hypertension. A previous research indicates that glycine consumption is linked to an increased risk of high blood pressure, possibly because glycine is predominantly found in animal proteins, and excessive meat consumption is a factor in elevated blood pressure (Stamler et al. 2013). Moreover, glycine plays a role in the formation of elastin, which regulates blood vessel elasticity, and changes in vascular elasticity represent another key mechanism in the development of hypertension (El Hafidi et al. 2006). It is noteworthy that prior research has shown that the metabolism of methionine can lead to the production of homocysteine, and elevated cysteine levels can induce the generation of vascular damaging factors, impairing endothelial function, and raising blood pressure (Li et al. 2024). Animal studies have demonstrated that early supplementation with D‐cysteine or L‐cysteine prevented hypertension and kidney injury in spontaneously hypertensive rats exposed to high salt intake by modulating oxidative stress and the renin‐angiotensin system (Hsu et al. 2018). Our findings suggest a potential association between oxidized cysteinylglycine (Cys‐gly, oxidized) and an increased risk of hyperaldosteronism. This is somewhat inconsistent with previous studies. According to existing literature, cysteine may regulate blood pressure through mechanisms such as inhibiting oxidative stress, increasing nitric oxide bioavailability, and improving insulin resistance. Moreover, cysteine is a component of glutathione, a tripeptide composed of glutamate and glycine, which has strong antioxidant properties and plays a role in blood pressure regulation (Vasdev et al. 2009). One possible explanation for the discrepancy is that hyperaldosteronism, as a form of secondary hypertension, may have pathophysiological mechanisms that differ from those of essential hypertension. Therefore, further research is needed to clarify the relationship between cysteine and hyperaldosteronism. In addition, our study identified several amino acid ratios that may be associated with the risk of hyperaldosteronism, including the ornithine to glutamate ratio, the alpha‐ketoglutarate to alpha‐ketobutyrate ratio, the histidine to alanine ratio, and the histidine to asparagine ratio. Currently, there is limited research on how different amino acid ratios may influence hyperaldosteronism. Future studies are warranted to further explore the roles of amino acids and their metabolic balance in the development of hyperaldosteronism.

Numerous previous studies have observed altered lipid profiles in patients with hyperaldosteronism. For instance, a meta‐analysis indicated that levels of triglycerides and low‐density lipoprotein (LDL) were higher in hyperaldosteronism patients compared to those with essential hypertension (Manosroi et al. 2022). Other studies have demonstrated a positive correlation between plasma LDL, high‐density lipoprotein (HDL), and plasma aldosterone concentrations, alongside a negative correlation between HDL and plasma cholesterol levels (Goodfriend et al. 1995; Hannich et al. 2018). However, observational studies can only provide correlations between lipids and hyperaldosteronism, and their results might be influenced by confounding factors such as environmental influences, dietary habits, and reverse causality. Using MR analysis, we identified several lipids that may have potential causal associations with hyperaldosteronism. Specifically, we found that 1‐linoleoylglycerol (18:2) levels, 3‐carboxy‐4‐methyl‐5‐pentyl‐2‐furanpropionate levels, PC levels, and palmitoleate (16:1n7) levels were suggestive risk factors for hyperaldosteronism. 3‐Carboxy‐4‐methyl‐5‐pentyl‐2‐furanpropionate is an endogenous furan fatty acid metabolite that influences lipid metabolism by modulating lipid metabolism pathways and participating in mitochondrial oxidative phosphorylation processes (Niwa 2013; Lai et al. 2023), which could also be a mechanism affecting hyperaldosteronism. PC is an oxidized low‐density lipoprotein, and current research on PC centers around the therapeutic role of anti‐PC antibodies in chronic inflammatory diseases such as type 2 diabetes, hypertension, and cardiovascular disease (Taleb et al. 2023). Studies have shown that antibodies against PC are negatively associated with various chronic inflammatory conditions in hospitalized versus non‐hospitalized obese subjects, with potential mechanisms involving anti‐inflammatory and immunomodulatory effects (Jujić et al. 2021). Given that hyperaldosteronism is a metabolic disease with pathogenesis similar to diabetes and hypertension, the above studies have suggested that PC promotes hyperaldosteronism, and anti‐PC antibodies could potentially become a strategy for treating metabolic diseases like hyperaldosteronism in the future, although further research is required to elucidate the exact mechanism by which PC impacts hyperaldosteronism. This is the first time we report the correlation between 1‐stearoyl‐2‐oleoyl‐GPE (18:0/18:1), citramalate, glycerol 3‐phosphate, palmitoleate (16:1n7) and hyperaldosteronism, and such associations have been rarely studied previously.

Amino acids and lipids are two major factors known to influence metabolic diseases. Our study suggests potential associations between certain amino acids and lipids and the risk of hyperaldosteronism. Nevertheless, we recognize that the relationships between metabolites and hyperaldosteronism are complex, which presents challenges for future research into the underlying mechanisms. Despite these limitations, the findings from our MR analysis may provide valuable insights and serve as a reference for further studies in this area.

Our study has several strengths. First, it is based on a comprehensive genetic exploration of the causal relationships between 1400 metabolites/metabolic ratios and hyperaldosteronism. The inclusion of a wide range of metabolites provides a relatively comprehensive overview of potential associations. Second, we applied four MR analysis methods along with sensitivity analyses, which helped reduce the influence of confounding factors and reverse causality compared to observational studies, thereby enhancing the objectivity and reliability of our findings. However, our study also has some limitations. First, the sample size of individuals with hyperaldosteronism was relatively limited, which may affect the stability of our results. Moreover, although all identified metabolites showed suggestive causal associations with hyperaldosteronism (*p* < 0.05), none met the Bonferroni‐corrected significance threshold (*p* < 3.57 × 10^−5^). Therefore, our findings should be interpreted with caution and are primarily of suggestive value. Second, while MR methods help explore potential causal relationships, the development of hyperaldosteronism is influenced by both genetic and environmental factors. Both genetic predisposition and environmental influences can affect the regulation of circulating metabolite levels, which may limit our understanding of the true causal relationships between metabolites and hyperaldosteronism. Third, our study population was restricted to individuals of European ancestry. Therefore, the findings may not be generalizable to other ethnic populations. Finally, this study focused solely on investigating the causal relationships between metabolites or metabolic ratios and hyperaldosteronism. Although we conducted pathway enrichment analysis and summarized the major metabolic pathways involved, we did not explore the underlying biological mechanisms in detail. Future studies, including well‐designed randomized controlled trials and basic research, are needed to further elucidate the functional roles of these metabolites in the pathogenesis of hyperaldosteronism.

## 5 Conclusion

In summary, our study identified 59 metabolites/metabolic ratios that showed suggestive causal associations with hyperaldosteronism. These metabolites may influence the development of hyperaldosteronism through amino acid and fatty acid metabolism pathways. From a genetic and metabolomic perspective, our findings provide preliminary evidence for the relationship between these metabolites and hyperaldosteronism.

## Author Contributions

**Chenyang Zhao:** conceptualization (equal), data curation (equal), funding acquisition (lead), investigation (equal), methodology (equal), validation (equal), writing – original draft (lead), writing – review and editing (lead). **Fangjun Chen:** conceptualization (equal), data curation (equal), validation (equal), writing – review and editing (equal). **Lixiu Peng:** writing – review and editing (equal). **Qiong Li:** conceptualization (equal), data curation (equal), formal analysis (lead), validation (equal), writing – review and editing (equal). **Yajing Pang:** data curation (equal), writing – review and editing (equal). **Chaoyan Yue:** conceptualization (equal), data curation (equal), formal analysis (lead), supervision (lead), validation (equal), visualization (equal), writing – review and editing (equal).

## Ethics Statement

Only publicly available GWAS data were used in this study, and the ethics approval and consent to participate could be available in the original GWAS study.

## Conflicts of Interest

The authors declare no conflicts of interest.

## Supporting information


Data S1.

## Data Availability

The summary data of IEU can be downloaded from the website https://gwas.mrcieu.ac.uk/. The summary data of FinnGen can be down loaded from the website https://www.finngen.fi/en/access_results.

## References

[fsn370532-bib-0001] Burgess, S. , A. Butterworth , and S. G. Thompson . 2013. “Mendelian Randomization Analysis With Multiple Genetic Variants Using Summarized Data.” Genetic Epidemiology 37, no. 7: 658–665. 10.1002/gepi.21758. 24114802 PMC4377079

[fsn370532-bib-0002] Burgess, S. , R. M. Daniel , A. S. Butterworth , and S. G. Thompson . 2015. “Network Mendelian Randomization: Using Genetic Variants as Instrumental Variables to Investigate Mediation in Causal Pathways.” International Journal of Epidemiology 44, no. 2: 484–495. 10.1093/ije/dyu176. 25150977 PMC4469795

[fsn370532-bib-0003] Burgess, S. , and S. G. Thompson . 2011. “Avoiding Bias From Weak Instruments in Mendelian Randomization Studies.” International Journal of Epidemiology 40, no. 3: 755–764. 10.1093/ije/dyr036. 21414999

[fsn370532-bib-0004] Chen, Y. , T. Lu , U. Pettersson‐Kymmer , et al. 2023. “Genomic Atlas of the Plasma Metabolome Prioritizes Metabolites Implicated in Human Diseases.” Nature Genetics 55, no. 1: 44–53. 10.1038/s41588-022-01270-1. 36635386 PMC7614162

[fsn370532-bib-0005] El Hafidi, M. , I. Pérez , and G. Baños . 2006. “Is Glycine Effective Against Elevated Blood Pressure?” Current Opinion in Clinical Nutrition and Metabolic Care 9, no. 1: 26–31. 10.1097/01.mco.0000196143.72985.9a. 16444815

[fsn370532-bib-0006] Erlic, Z. , P. Reel , S. Reel , et al. 2021. “Targeted Metabolomics as a Tool in Discriminating Endocrine From Primary Hypertension.” Journal of Clinical Endocrinology and Metabolism 106, no. 4: 1111–1128. 10.1210/clinem/dgaa954. 33382876 PMC7993566

[fsn370532-bib-0007] Fallo, F. , C. Pilon , and R. Urbanet . 2012. “Primary Aldosteronism and Metabolic Syndrome.” Hormone and Metabolic Research = Hormon‐Und Stoffwechselforschung = Hormones et Metabolisme 44, no. 3: 208–214. 10.1055/s-0031-1295412. 22116746

[fsn370532-bib-0008] Fallo, F. , F. Veglio , C. Bertello , et al. 2006. “Prevalence and Characteristics of the Metabolic Syndrome in Primary Aldosteronism.” Journal of Clinical Endocrinology and Metabolism 91, no. 2: 454–459. 10.1210/jc.2005-1733. 16291704

[fsn370532-bib-0009] Flores‐Guerrero, J. L. , D. Groothof , M. A. Connelly , J. D. Otvos , S. J. L. Bakker , and R. P. F. Dullaart . 2019. “Concentration of Branched‐Chain Amino Acids Is a Strong Risk Marker for Incident Hypertension.” Hypertension 74, no. 6: 1428–1435. 10.1161/hypertensionaha.119.13735. 31587574

[fsn370532-bib-0010] Funder, J. W. , R. M. Carey , F. Mantero , et al. 2016. “The Management of Primary Aldosteronism: Case Detection, Diagnosis, and Treatment: An Endocrine Society Clinical Practice Guideline.” Journal of Clinical Endocrinology and Metabolism 101, no. 5: 1889–1916. 10.1210/jc.2015-4061. 26934393

[fsn370532-bib-0011] Goodfriend, T. L. , B. Egan , K. Stepniakowski , and D. L. Ball . 1995. “Relationships Among Plasma Aldosterone, High‐Density Lipoprotein Cholesterol, and Insulin in Humans.” Hypertension 25, no. 1: 30–36. 10.1161/01.hyp.25.1.30. 7843750

[fsn370532-bib-0012] Greco, M. F. , C. Minelli , N. A. Sheehan , and J. R. Thompson . 2015. “Detecting Pleiotropy in Mendelian Randomisation Studies With Summary Data and a Continuous Outcome.” Statistics in Medicine 34, no. 21: 2926–2940. 10.1002/sim.6522. 25950993

[fsn370532-bib-0013] Hannich, M. , H. Wallaschofski , M. Nauck , et al. 2018. “Physiological Aldosterone Concentrations Are Associated With Alterations of Lipid Metabolism: Observations From the General Population.” International Journal of Endocrinology 2018: 4128174. 10.1155/2018/4128174. 29780416 PMC5892232

[fsn370532-bib-0014] Hanslik, G. , H. Wallaschofski , A. Dietz , et al. 2015. “Increased Prevalence of Diabetes Mellitus and the Metabolic Syndrome in Patients With Primary Aldosteronism of the German Conn's Registry.” European Journal of Endocrinology 173, no. 5: 665–675. 10.1530/eje-15-0450. 26311088

[fsn370532-bib-0015] Hsu, C. N. , Y. J. Lin , P. C. Lu , and Y. L. Tain . 2018. “Early Supplementation of d‐Cysteine or l‐Cysteine Prevents Hypertension and Kidney Damage in Spontaneously Hypertensive Rats Exposed to High‐Salt Intake.” Molecular Nutrition & Food Research 62: 1700596. 10.1002/mnfr.201700596. 28981205

[fsn370532-bib-0016] Jujić, A. , J. Korduner , H. Holm , et al. 2021. “Antibodies Against Phosphorylcholine in Hospitalized Versus Non‐Hospitalized Obese Subjects.” Scientific Reports 11, no. 1: 20246. 10.1038/s41598-021-99615-z. 34642415 PMC8511239

[fsn370532-bib-0017] Kittithaworn, A. A. , P. Dogra , J. Saini , et al. 2024. “Enhanced Chronic Inflammation and Increased Branched Chain Amino Acids in Adrenal Disorders: A Cross‐Sectional Study.” Journal of Clinical Endocrinology and Metabolism 110: e330–e338. 10.1210/clinem/dgae204. PMC11747673 38546526

[fsn370532-bib-0018] Knuchel, R. , Z. Erlic , S. Gruber , et al. 2024. “Association of Adrenal Steroids With Metabolomic Profiles in Patients With Primary and Endocrine Hypertension.” Frontiers in Endocrinology 15: 1370525. 10.3389/fendo.2024.1370525. 38596218 PMC11002274

[fsn370532-bib-0019] Lai, Q. , X. Zhu , L. Zhang , et al. 2023. “Inhibition of OAT1/3 and CMPF Uptake Attenuates Myocardial Ischemia‐Induced Chronic Heart Failure via Decreasing Fatty Acid Oxidation and the Therapeutic Effects of Ruscogenin.” Translational Research: The Journal of Laboratory and Clinical Medicine 261: 1–15. 10.1016/j.trsl.2023.06.001. 37315712

[fsn370532-bib-0020] Lawlor, D. A. , R. M. Harbord , J. A. Sterne , N. Timpson , and G. D. Smith . 2008. “Mendelian Randomization: Using Genes as Instruments for Making Causal Inferences in Epidemiology.” Statistics in Medicine 27, no. 8: 1133–1163. 10.1002/sim.3034. 17886233

[fsn370532-bib-0021] Li, W. , D. Chen , S. Y. Wong , M. P. Kwan , and L. A. Tse . 2024. “Associations of Smoking Status With Carotid Atherosclerosis: Mediated Role of Blood Indexes and Blood Pressure.” Nutrition, Metabolism, and Cardiovascular Diseases 35: 103709. 10.1016/j.numecd.2024.08.003. 39271389

[fsn370532-bib-0022] Manosroi, W. , P. Phudphong , P. Atthakomol , and M. Phimphilai . 2022. “The Differences of Serum Lipid Profiles Between Primary Aldosteronism and Essential Hypertension: A Meta‐Analysis and Systematic Review.” BMC Endocrine Disorders 22, no. 1: 217. 10.1186/s12902-022-01135-y. 36045354 PMC9429522

[fsn370532-bib-0023] Niwa, T. 2013. “Removal of Protein‐Bound Uraemic Toxins by Haemodialysis.” Blood Purification 35, no. Suppl 2: 20–25. 10.1159/000350843. 23676831

[fsn370532-bib-0024] Ruiz‐Canela, M. , E. Toledo , C. B. Clish , et al. 2016. “Plasma Branched‐Chain Amino Acids and Incident Cardiovascular Disease in the PREDIMED Trial.” Clinical Chemistry 62, no. 4: 582–592. 10.1373/clinchem.2015.251710. 26888892 PMC4896732

[fsn370532-bib-0025] Sekula, P. , M. F. Del Greco , C. Pattaro , and A. Köttgen . 2016. “Mendelian Randomization as an Approach to Assess Causality Using Observational Data.” Journal of the American Society of Nephrology 27, no. 11: 3253–3265. 10.1681/asn.2016010098. 27486138 PMC5084898

[fsn370532-bib-0026] Skrivankova, V. W. , R. C. Richmond , B. A. R. Woolf , et al. 2021. “Strengthening the Reporting of Observational Studies in Epidemiology Using Mendelian Randomization: The STROBE‐MR Statement.” JAMA 326, no. 16: 1614–1621. 10.1001/jama.2021.18236. 34698778

[fsn370532-bib-0027] Stamler, J. , I. J. Brown , M. L. Daviglus , et al. 2013. “Dietary Glycine and Blood Pressure: The International Study on Macro/Micronutrients and Blood Pressure.” American Journal of Clinical Nutrition 98, no. 1: 136–145. 10.3945/ajcn.112.043000. 23656904 PMC3683815

[fsn370532-bib-0028] Taleb, A. , P. Willeit , S. Amir , et al. 2023. “High Immunoglobulin‐M Levels to Oxidation‐Specific Epitopes Are Associated With Lower Risk of Acute Myocardial Infarction.” Journal of Lipid Research 64, no. 6: 100391. 10.1016/j.jlr.2023.100391. 37211249 PMC10275726

[fsn370532-bib-0029] Vasdev, S. , P. Singal , and V. Gill . 2009. “The Antihypertensive Effect of Cysteine.” International Journal of Angiology: Official Publication of the International College of Angiology, Inc 18, no. 1: 7–21. 10.1055/s-0031-1278316. 22477470 PMC2721729

[fsn370532-bib-0030] Würtz, P. , P. Soininen , A. J. Kangas , et al. 2013. “Branched‐Chain and Aromatic Amino Acids Are Predictors of Insulin Resistance in Young Adults.” Diabetes Care 36, no. 3: 648–655. 10.2337/dc12-0895. 23129134 PMC3579331

